# Dataset of verbal evaluation of umami taste in Europe

**DOI:** 10.1016/j.dib.2019.105102

**Published:** 2020-01-07

**Authors:** Emilia Iannilli, Antti Knaapila, Maria Paola Cecchini, Thomas Hummel

**Affiliations:** a Interdisciplinary Center “Smell & Taste”, Department of Otorhinolaryngology, TU Dresden, Germany; b Department of Food and Nutrition, University of Helsinki, Finland; c Department of Biochemistry, University of Turku, Finland; d Department of Neurosciences, Biomedicine and Movement Sciences, Section of Anatomy and Histology, School of Medicine, University of Verona, Italy

**Keywords:** Umami perception, Familiarity, Taste, Flavors, Food palatability, Salt, Synergism, Glutamate

## Abstract

The data presented here includes verbal descriptors used by Finnish, German and Italian subjects to express the quality of an umami taste solution offered in a blind fashion. The dataset refers to the research article “A cross-cultural survey of Umami Familiarity in European Countries” [1]. Data shows that a total of 106 different classes of words, including synonyms, were used by the Finnish group, 64 different classes of words, including synonyms, were used by the German group, and a total of 70 different classes of words, including synonyms, were used by the Italian group. The descriptors are reported in Excel tables and visualized in a bar graph where the length of the bars indicates the number of given answers for each class.

**Table:** Specifications Table

Subject area	*Biology, Psychology*
More specific subject area	*Food preference in a cross-cultural survey*
Type of data	*Tables, graphs*
How data was acquired	*A survey was presented with a written questionnaire accompanied by a practical gustatory experience of the taste samples. The survey was collected at the entrance of museums, galleries, hospitals, private companies, pharmacies, educational institutes and auditoriums. The volunteers were driven by personal interest in the scientific significance of the research subject.*
Data format	*Raw*
Experimental factors	*The verbal descriptions for the Umami taste were grouped in one class if the semantic descriptor had the same meaning. For example in Italian the descriptors “buono” and “gustoso” (meaning: good, tasty) were embedded in one single class.*
Experimental features	*The data here reported, represent a vocabulary resource of Umami semantic descriptors in three languages, Finnish, German and Italian.*
Data source location	*Three different European Countries were included: Finland, Germany and Italy.*
Data accessibility	*Data are directly accessible with this article.*
Related research article	*Cecchini, Maria Paola, Knaapila, Antti, Hoffmann, Eileen, Federico, Boschi, Hummel, Thomas and Iannilli, Emilia, A cross-cultural survey of Umami Familiarity in European Countries. Food Quality and Preference (FQAO_2018_605_R2).*

**Table:** 

**Value of the Data** • Although all sorts of foods are rich in umami taste, Europeans still have difficulty recognizing it. • This data collects the verbal expressions that people across European countries have used to describe the Umami taste in a survey. • The dataset is valuable in several perspectives: to guide food preference and choice for fields like nutrition, and in the food industry • It is important for comprehension of the familiarity of these populations with the Umami taste for future development of clinical tests used to detect gustatory dysfunctions. These tests in Europe are commonly limited only to sweet, salt, sour and bitter tastes.

## 1 Data

**Fig. 1:**
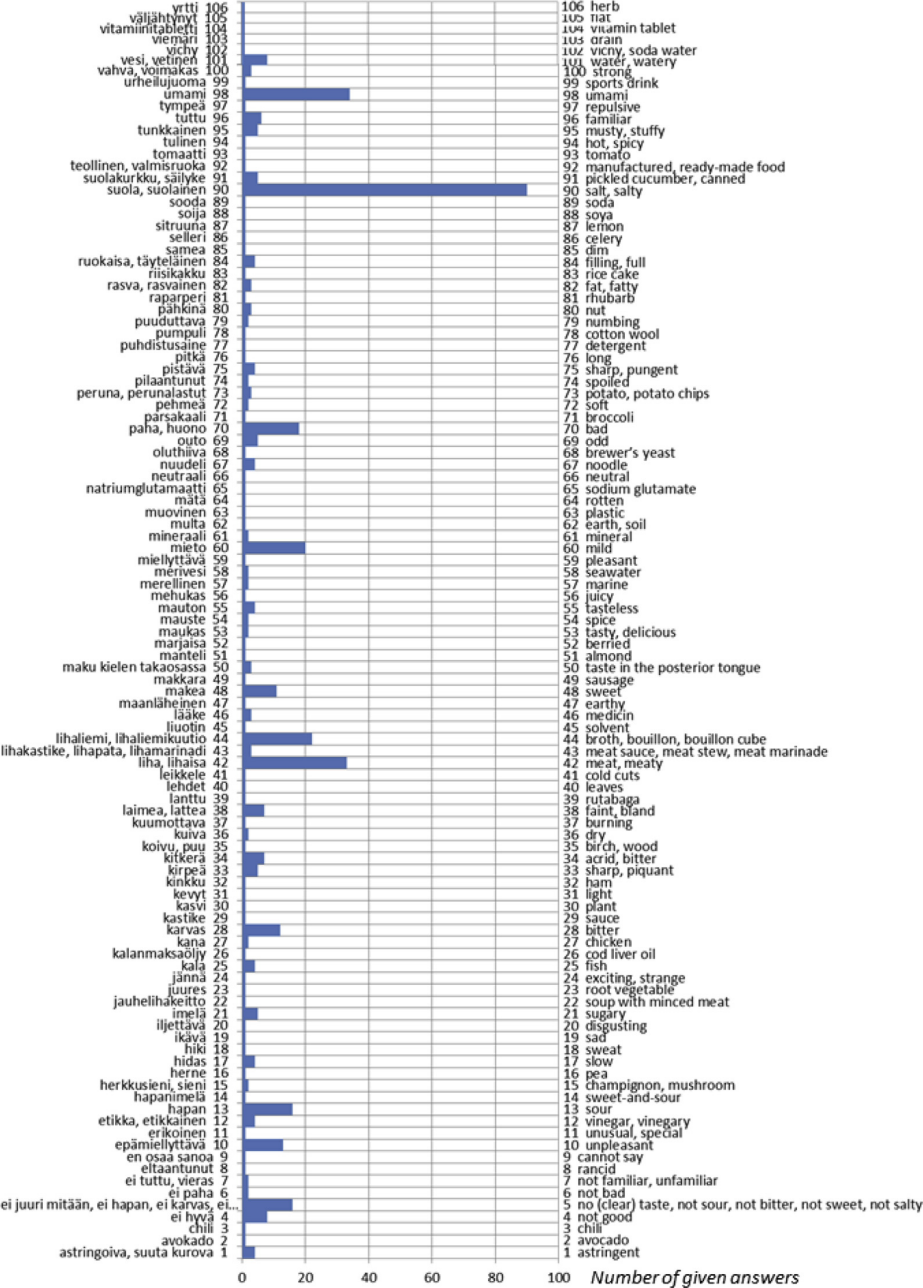
Verbal descriptors used by the Finnish subjects to express the umami taste solution (Finnish original words are reported on the left axis, English translation is reported on the right axis). A total of 106 different classes of words, including synonyms, were used. The length of the bars indicates the number of given answers for each class.

**Fig. 2:**
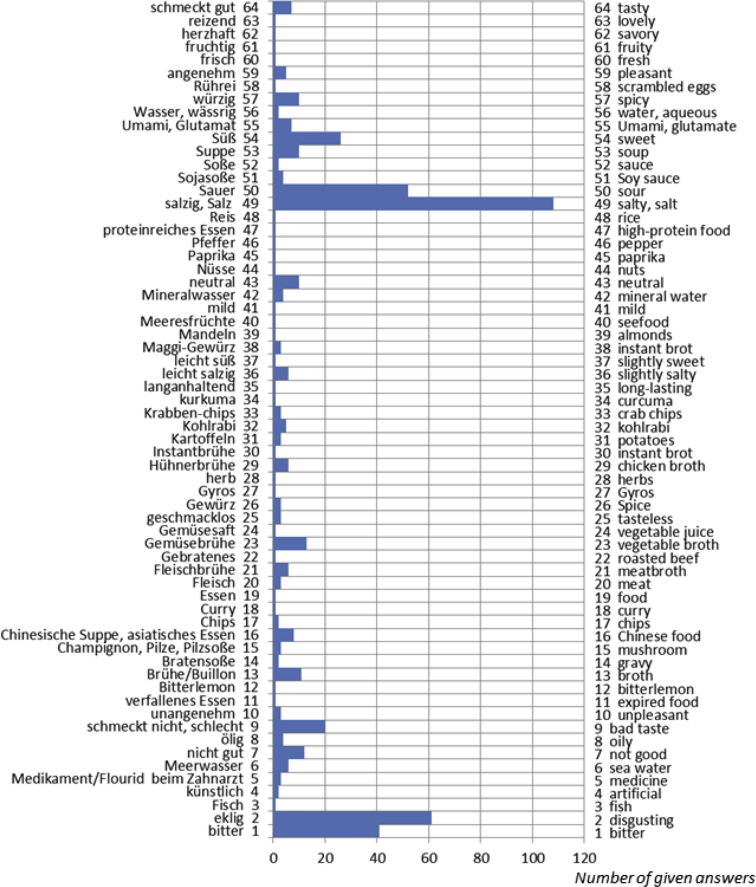
Verbal descriptors used by the German subjects to express the umami taste solution (German original words are reported on the left axis, English translation is reported on the right axis). A total of 64 different classes of words, including synonyms, were used. The length of the bars indicates the number of given answers for each class.

**Fig. 3:**
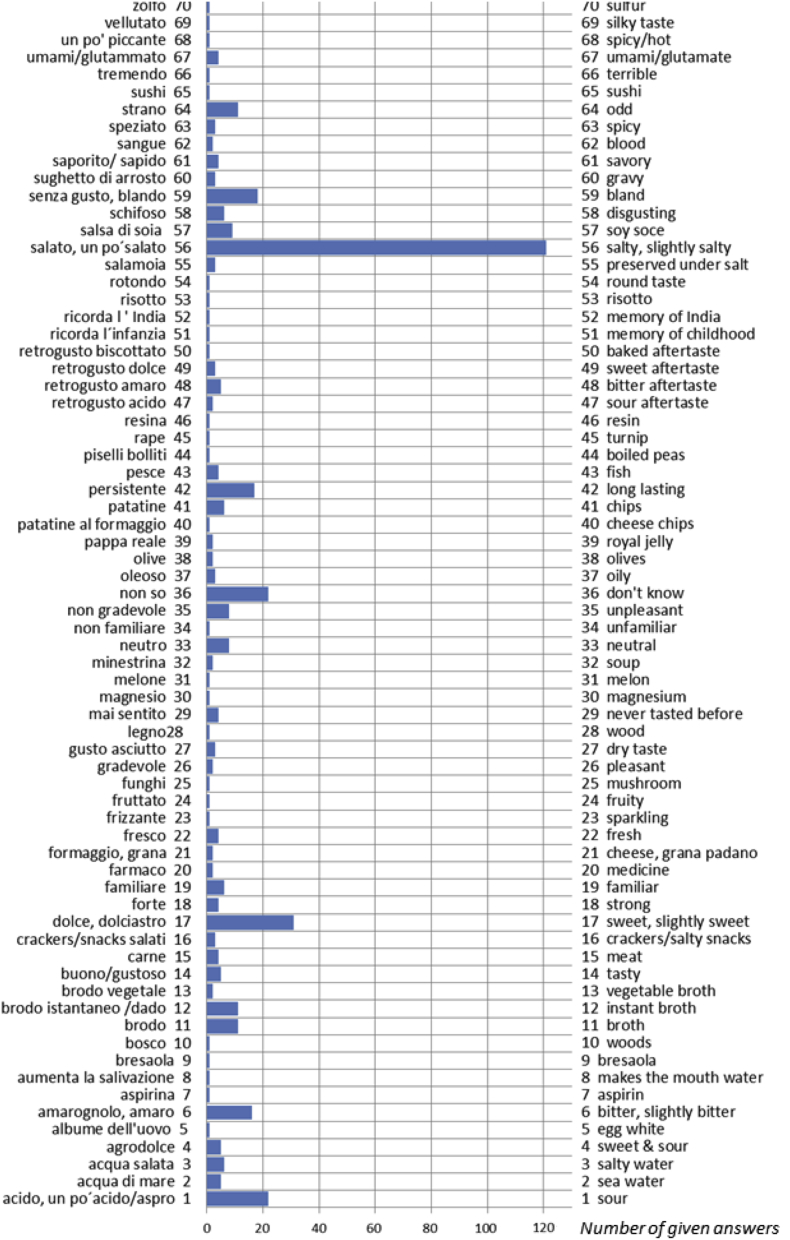
Verbal descriptors used by the Italian subjects to express the umami taste solution (Italian original words are reported on the left axis, English translation is reported on the right axis). A total of 70 different classes of words, including synonyms, were used. The length of the bars indicates the number of given answers for each class.

The dataset contains verbal descriptors spontaneously expressed from volunteers just after the tasting of an Umami solution, without any clue of the taste. The data was collected in three different countries in Europe: Finland, Germany and Italy. Fig. 1, Fig. 2 and Fig. 3 represent the bar graph of data classified in categories both in the original language and translated in English. The raw data is available as supplementary material in three Excel tables for the Finnish, the German and the Italian groups respectively.

## 2 Experimental design, materials and methods

### 2.1 Participants

The data is based on a social experiment where the lexical descriptors were collected after the volunteer tasted an unlabeled odorless and colorless liquid solution. 300 volunteers from Finland, 271 from Germany and 252 from Italy participated after been asked in public areas like museums, galleries, hospitals, private companies, pharmacies, educational institutes and auditoriums. Details about the demography of the groups are reported in Ref. [1].

### 2.2 Solution and experimental design

**Fig. 4:**
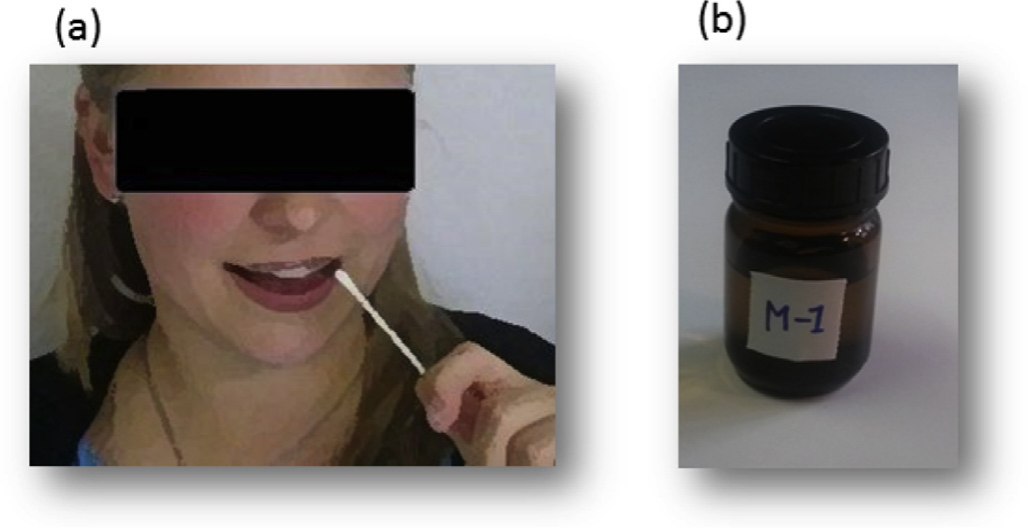
Panel (a) A cleaned cotton swab was soaked in the solution and offered to the volunteers to taste it. Panel (b) the umami liquid solution was presented in dark, glass jar; the jar was labeled with an obscure code to the subjects.

Monosodium-glutamate (MSG) was diluted in water for injectable solution at the concentration of 241mM. The concentrations was based on a previous research [2] that ensured it to be supra-threshold and easily perceivable by a healthy subject. The umami liquid solution was presented in a dark, glass jar, so that subjects were blind towards the content of the jar. As a general procedure, a sterilized cotton swab, soaked in one of the solution, was offered to the subject who had to put it into their mouth and suck on it to perceive the taste (Fig. 4). Before placing each cotton bud sample into the mouth, the subject was asked to rinse their mouth with a sufficient amount of water. The experimenter offered the sample to the volunteer and asked them to describe the taste with their own words. The verbal descriptors were registered by the experimenter in the original language on the survey sheet and later transferred to an Excel file.

### 2.3 Word classification criteria and procedure

A classification, generated at a later time by the respective mother tongue investigator, was performed based on (a) the closely related semantic meaning of the descriptors, (b) a common cultural practice and (c) a more general experience not only related to food. Synonyms, similar words and analogous concepts or meaning were grouped in the same category. An example of synonyms or similar words is the German words “Umami” and “Glutamat”, respectively “Umami” and “glutamate” in English.

An example of analogous common concept in the Finnish group are the words “lihakastike”, “lihapata” or “lihamarinadi” (respectively in english “meat sauce”, “meat stew” and “meat marinade”) that in Finnish express the same concept of a long cooked meat in a base of herbs and aromas, although in other culture marinate may indicate a cold and long soaking process. In the same line the experimenter was also asked to take into account cultural food experience that my variate among the countries even if the semantic meaning indicates similar products. For example in German “Gemüsesaft” and “Gemüsebrühe”, respectively in English “vegetable juice” and “vegetable broth”, while they physically represent a similar product, which is a vegetable pure, in the common German experience the vegetable juice is more like a fruit juice, doesn't contain salt and often is mixed with fruits too, on the contrary the vegetable broth contain salt as well as other savory aromas.

Finally, descriptors indicating a generally bad or good experience not directly related to food were also taken separately. An example of this are the three classes of Italian descriptors: “tremendo”, “schifoso”, and “non-gradevole”, respectively in English “terrible”, “disgusting” and “unpleasant”. Although they have the same meaning in English, in Italian they have different conceptual association. With “tremendo” it is usually referred to a general experience, not specifically associated with food. “Schifoso” in a context of taste is very specific for food and finally “non-gradevole” is a descriptor that has less strength than “schifoso”.

There was no limit to how many word classes could be assigned to a single response.
